# Maternity experiences of mothers with multiple disadvantages in England: A qualitative study

**DOI:** 10.1016/j.wombi.2018.05.009

**Published:** 2019-04

**Authors:** Jenny McLeish, Maggie Redshaw

**Affiliations:** Policy Research Unit in Maternal Health and Care, National Perinatal Epidemiology Unit, Nuffield Department of Population Health, University of Oxford, Old Road Campus, Headington, Oxford OX3 7LF, UK

**Keywords:** Pregnant women, Vulnerable populations, Midwifery, Disadvantaged, Maternal health services

## Abstract

**Background:**

Disadvantaged mothers and their babies are at increased risk of poor perinatal outcomes and have less positive experiences of maternity care.

**Aim:**

To explore the maternity care experiences of mothers with multiple disadvantages.

**Methods:**

A qualitative descriptive study based on semi-structured interviews with 40 mothers with multiple disadvantages, using thematic analysis.

**Findings:**

Four themes emerged: ‘A confusing and frightening time’, ‘Longing to be respected as an individual’, ‘The importance of choice and control’, and ‘Needing trust to feel safe’. Mothers brought feelings of powerlessness and low self-esteem to their encounters with maternity professionals, which could be significantly worsened by disrespectful care. They needed support to navigate the complex maternity system. Positive experiences were much more likely where the mother had received continuity of care from a specialist midwife or small team.

**Discussion and conclusion:**

Mothers with multiple disadvantages value being treated as an individual, making informed choices, and feeling safe, but they may lack the confidence to ask questions or challenge disrespectful treatment. Training and supervision should enable maternity professionals to understand how confusing maternity care can be to very disadvantaged mothers. It should emphasise the need to provide accessible and empowering information and guidance to enable all mothers to make choices and understand the system. Leaders of maternity services need to do more to challenge negative staff attitudes and ensure that that all mothers are treated at all times with kindness, respect and dignity. Specialist midwives can deliver a high quality service to mothers experiencing multiple disadvantages.

**Statement of significance**

**Problem or issue**

Women have the right to empowering, woman-centred, respectful maternity care based on informed choice.

**What is already known**

Specific groups of disadvantaged women are less likely to report positive experiences of maternity care in national surveys and qualitative studies.

**What this paper adds**

This paper reports on the maternity experiences of mothers experiencing multiple disadvantages, defined as low socio-economic status and at least one additional factor – under 25, recent migrants, asylum seekers and refugees, from Black and ethnic minority communities, single parents, living with physical or mental illness, and domestic abuse. These voices are rarely heard in research.

## 1 Introduction

Maternity policy in England is built on the core principles of the right of each mother to make informed choices about her care, and to be treated with kindness, respect and dignity. Care should be compassionate and woman-centred, tailored to her individual needs and delivered in a way that enhances her experience and enables her to remain in control.^1^, ^2^ National surveys have found that these aspirations are met for the majority of service users, for example in 2014 two thirds of women felt that they were always involved in decisions about their care during labour and birth, and around nine in ten felt they were always treated with respect and kindness.^3^

Disadvantaged mothers are less likely to respond to surveys, and those who respond are less likely to report positive experiences of maternity care.^3^ In the national maternity surveys, mothers from Black, Asian and Minority Ethnic (BAME) communities were less likely to feel always treated respectfully or always involved enough in decisions; single mothers were less likely to always be involved in decisions and to be satisfied with their care; women from poor backgrounds were less likely to feel always treated respectfully or spoken to in a way they could understand; and women with physical and learning disabilities were less likely to feel involved in decisions about their care, to be treated with respect and to be spoken to in a way they could understand.^3^, ^4^, ^5^, ^6^

Qualitative research with specific groups of disadvantaged mothers has also found patterns of poor experiences of maternity care. For example, mothers from BAME and migrant communities have reported poor communication, lack of respect for cultural needs, poor management of female genital mutilation and prejudiced staff attitudes.^7^, ^8^, ^9^, ^10^ Young mothers have felt disempowered and stigmatised.^11^ Mothers with mental health problems have felt lost in the system, neglected or over-scrutinised, and misunderstood.^12^ Some mothers with learning disabilities have experienced poor communication, inaccessible information, and denial of choice.^13^, ^14^ Mothers with physical disabilities have encountered problems with physical access to facilities, poor staff awareness, and discriminatory attitudes.^15^

Disadvantaged mothers and their babies are also at increased risk of poor physical and mental health outcomes, specifically if mothers are poor, migrants, from BAME communities, single, or young.^16^, ^17^, ^18^, ^19^, ^20^, ^21^ National guidance recommends making maternity care and information more accessible for pregnant women who have ‘complex social factors’,^22^ but it is not known to what extent this ambition is met. This study therefore builds on the existing literature focused on individual aspects of social complexity and aims to explore the maternity experiences of mothers each of whom was vulnerable through multiple challenges. Most significantly it was possible in this interview-based study, working with a range of organisations supporting disadvantaged mothers, to hear the voices and views of women not easily accessed by conventional survey methods.

## 2 Participants, ethics and methods

### 2.1 Study design

This was a qualitative descriptive study,^23^ based on semi-structured, in-depth interviews, theoretically informed by phenomenological social psychology.^24^ This “low-inference” design^23^ enables mothers’ voices to be heard while acknowledging the role of both participants’ understandings and the researchers’ interpretations in the production of knowledge.^25^ The Oxford University Medical Sciences Research Ethics Committee (reference MSD-IDREC-C1-2013-111) approved the study.

### 2.2 Participants

Participants were eligible for this study if they met the criteria of (1) having had a baby in the UK in the last five years and (2) experiencing multiple disadvantages, defined as low socio-economic status and at least one additional factor – aged under 25, recent migrants, asylum seekers and refugees, mothers from BAME communities, single parents, living with physical or mental illness, and domestic abuse. Low socio-economic status was defined as low paid work, relying on unemployment or disability benefits, having no recourse to public funds, being homeless or in a temporary hostel, and destitution.

Forty disadvantaged mothers were included in this study. 24 were first time mothers and 16 had between two and five children (mean 3.1). Of the multiparous mothers, for seven this was their first birth in the UK. All of the babies were born in hospital.

All participants were socio-economically disadvantaged, with 29 being severely disadvantaged (defined as dependent on unemployment or disability benefits, without recourse to public funds, homeless, living in a temporary hostel, and/or destitute), and 11 being moderately disadvantaged (defined as having a partner in low paid work). All experienced between one and five additional factors reflecting vulnerability: being under 25, a recent migrant, an asylum seeker or refugee, from British BAME communities, a single parent, having a physical health condition, having mental health difficulties, and domestic abuse. These factors (mean 4.2 per participant) are shown in Table 1. In addition, some participants had experienced traumatic situations including rape, torture, the death of a child or partner, being the victim of people-trafficking, being held in immigration detention, and having children removed from their care.

**Table 1:** Characteristics of women interviewed.

	Number	%
Socio-economic factors		
Severe socio-economic disadvantage	29	72.5
Moderate socio-economic disadvantage	11	27.5
Single parent	24	60.0
Under 25 years of age	10	25.0
Domestic abuse	6	15.0

Ethnicity and migration status		
Born outside the UK	28	70.0
Black African	18	45.0
Asian	3	7.5
White (Eastern Europe)	3	7.5
Other	4	10.0
Asylum seeker/refugee^a^	16	40.0
Born in the UK	12	30.0
Asian British	1	2.5
Black British	3	7.5
White British	8	20.0

Health		
Long term health condition or disability	11	27.5
Poor mental health	27	67.5
Low mood/anxiety	20	50.0
Receiving treatment for severe mental illness	7	17.5

Number of characteristics reflecting vulnerability per woman		
2	4	10.0
3	9	22.5
4	8	20.0
5	12	30.0
6	7	17.5

a Including women whose asylum claim had been refused.

### 2.3 Data collection

The participants were recruited as part of a wider study exploring perinatal peer support and maternity care for disadvantaged mothers, through ten third sector projects offering peer support to mothers during pregnancy and after birth.^26^ These projects were chosen to reflect a diversity of geographical locations in England (in Bradford, Bristol, Burnley, Huddersfield, Halifax, Hull, London and rural North Yorkshire), and target populations (mothers with very complex needs, young mothers, South Asian mothers, refugee and asylum seeker mothers, mothers living with HIV, mothers with mental illness, disadvantaged local mothers).

The researcher met the co-ordinator of each project to introduce the research. The co-ordinators then described the research to supported mothers and peer supporters using the study information leaflets and either asked permission for the researcher to contact them, or arranged with those who wished to participate a time for interview. Two potential participants decided not to participate when contacted by the researcher. The researcher had not previously had contact with any of the participants.

In-depth qualitative semi-structured interviews were carried out between July 2013 and March 2015. Interviews were face to face at a location chosen by the participant, with the exception of one interview carried out by telephone at the participant’s request. Written informed consent was obtained before each interview (for the telephone interview, informed consent was obtained orally and recorded in writing). Interviews with supported mothers explored experiences of receiving peer support and experiences of using the maternity services, specifically what they felt about the maternity professionals, information-giving, and making informed choices. The topic guide for peer supporter interviews focused on their experiences of giving support to other women and did not ask about their maternity experiences. However, some peer supporters who were themselves disadvantaged mothers spontaneously spoke about their own recent maternity experiences, and the interviewer then explored similar topics to those in the supported mothers’ interviews. The duration of interviews varied (range 16–90 min, median 44 min); the shorter length of a few interviews was due to mothers needing to attend to their young children. Although professional interpreting for participants whose first language was not English was offered, none took up the offer, but at the interviewee’s request one interview was informally interpreted by a peer supporter. All the interviews were audio-recorded and professionally transcribed.

Sampling for the wider study was purposive insofar as all participants had experience of giving or receiving perinatal peer support. Within that group (n = 100), all who met the inclusion criteria for this study (n = 40) were included in this analysis. 35 women (given identifiers M01–M35) had received peer support and five (given identifiers M36–M40) were peer supporters.

### 2.4 Data analysis

Interviews were analysed using inductive thematic analysis.^27^ Transcripts were first checked against the audio recording, and then read and reread, and codes were identified inductively and recorded using NVIVO software. Codes were refined, combined and disaggregated as data collection continued, and emergent themes identified; earlier codes and emergent themes were reconsidered in the light of subsequent interviews. To enhance the validity of the analysis, one researcher undertook thematic analysis of all the transcripts and the other analysed a subset. Codes and emerging themes were discussed and agreed. Both researchers were aware of the need to approach the analysis reflexively, putting aside their existing knowledge of the topic so that the analysis remained close to participants’ accounts, and acknowledging the potential impact of their own perspectives as White, UK-born women with children. The impact of peer support on women’s experiences of maternity care was also analysed and has been reported separately.^26^

## 3 Findings

Four themes emerged from the analysis of maternity care experiences: ‘*A confusing and frightening time*’, ‘*Longing to be respected as an individual*’, ‘*The importance of choice and control*’, and ‘*Needing trust to feel safe*’. Illustrative quotations from these themes are shown in Table 2.

**Table 2:** Themes with illustrative quotations.

Themes	Positive experiences	Negative experiences
A confusing and frightening time	“*I ask my midwife and she was really helpful so I knew what to expect.*”	“*I didn’t know anything…what midwife want from me.*”
Longing to be respected as an individual	“*[She] actually thought about me as a person, rather than just being a pregnant mum.*”	*“They were treating me like I was stupid… making you feel like you weren’t good enough.”*
The importance of choice and control	“*They gave me many choice… everything about that they explained to me.*”	“*She said, ‘...The doctor is the one who is going to decide.’*”
Needing trust to feel safe	*“They saved my life.”*	“*I were convinced they were going to kill me.*”

### 3.1 A confusing and frightening experience

For many of the mothers, the maternity services represented a complex system which they did not understand: “*I didn’t know anything, I don’t know how I born him or how I went to my appointments…which hospital I give birth, what midwife want from me*” (M27). Lacking guidance, they struggled to understand how to make use of the services:

“*I didn’t know who the midwives was and I didn’t know how things work… I didn’t know what was going on...I wasn’t sure what was happening...I didn’t have a midwife or someone to support me and explain to me things or what to do.*” (M17)

For some migrant women this was because the system was so different from what they had experienced in their home countries or because there were language obstacles: “*I could not understand them*” (M18). For other first time mothers it was all a new experience and they felt “*scared*” (M35)*,* “*frightened*” (M17) or “*panicking*” (M15) about unfamiliar aspects of maternity care. Many did not feel able to ask questions because the midwives appeared too busy: “*I had questions which my midwives were not giving me answers to. You meet your midwife for 10/15* *minutes and all she says is, ‘*Don’t worry, [the baby]’s going to be fine*’*” (M04). Others lacked confidence to ask questions because they were not sure where professional boundaries lay and did not see the midwives as approachable:

“*Because of my sickness they put me in the red group and I don’t know what’s the meaning [of] red group…I’m scared if I talk about my feeling they [will] say, ‘Okay, that is not our job’*” (M27).

One consequence of this lack of information was that mothers were left to fill the gap with their own “*weird imaginations*” (M04), making them feel even less confident and more afraid: “*I was thinking, ‘Oh no, how big’s this [caesarean] cut going to be?’ And I was imagining they were going to proper slice you open*” (M15). Others had relied on their community for information: “*[My relative] told me of these horror stories about what it’s like and how they don’t look after you… I was so anxious about going to the hospital*” (M14).

Two mothers had delayed starting maternity care because their immigration status meant they were not eligible for free National Health Service (NHS) care, and they were given no clear information about their rights. One had sought an abortion but by the time she had been referred between four different organisations, she had passed the time limit: “*The doctor say she can’t give me no letter anymore to do abortion… because then I was big already*” (M11). The other mother had been unable to register with a GP and was not told that she could access maternity care directly:

“*They said I have to register and I have to bring documents to prove I am entitled to NHS and because I didn’t have it I just hide, went back into my shell and I was just there and not knowing what to do… I didn’t even know I was having twins until I was 20 weeks pregnant before I saw the midwife for the first time*”. (M22)

By contrast, a quarter of mothers were under the care of a specialist midwife or midwifery team – roles specifically dedicated to women with social complexity or physical or mental health conditions, and characterised by a deeper knowledge of the issues and increased continuity of care. These mothers had little difficulty navigating the unfamiliar system because they were guided through it: “*I ask my midwife and she was really helpful so I knew what to expect*” (M06).

### 3.2 Longing to be respected as an individual

Some mothers described individual midwives very positively, for example “*lovely*” (M38), “*nice*” (M16, M19, M34), “*so gentle and so supportive*” (M12). Sometimes these positive descriptions related to the midwives’ attitudes, for example one mother was delighted to be treated without discrimination: “*They didn’t treat me like I was less than anybody else. Yeah, they treat me like everybody’s equal*” (M09). For others they were based on unanticipated and appreciated aspects of care, for example, home visits: “*They came my place and they were so kind*” (M10). Most commonly they reflected encounters with midwives who had demonstrated that they saw and cared about the mother as an individual: “*They would just make you feel completely reassured and looked after and respected*” (M29).

Some midwives had done this by giving the mother their full attention during appointments: “*They really listened…they didn’t look at the watch and just hurry up*” (M06). One mother recalled attentive care from the entire obstetric team: “*They made me feel special*” (M028). Other midwives had taken an interest in the multiple problems mothers faced: “*they worried about me*” (M02), and assisted them by making referrals: “S*he was all along the nicest midwife ever. She helped me a lot and she direct me to [support organisation]*” (M07).

Mothers who had received specialist care were particularly likely to feel individually acknowledged, understood, and valued: “*[The specialist midwife] actually thought about me as a person, rather than just being a pregnant mum*” (M05). Specialist midwives were also particularly likely to be able to support mothers in crisis situations effectively: “*The midwife is the first person that ever helped me out*” (M03). Several extremely vulnerable mothers indicated that specialist professionals had built up a relationship with them, which had an enormous impact on their experience and their ability to trust: “*She is like the best consultant in the whole world...She’s not just concerned about [the] medical you… she is just somebody I could talk to about anything at all*” (M26). For a few mothers, continuity of care was not important if they were consistently treated with kindness: “*I had loads of different [midwives]. But I found they were all nice*” (M19).

Half of the mothers described negative experiences of interactions with staff. Some felt processed through a system by professionals who followed procedures without really noticing the woman in front of them, so that mothers were made to feel “*just a kind of hospital routine*” (M23). These mothers described midwives as lacking warmth: “*very formal*” (M27), “*technical*” (M04), “*abrupt*” (M14), and interacting “*like they had a list, and the things they had to tick and check and that’s it*” (M06). Others had experienced unpleasant and disrespectful attitudes, for example: *“they see us a low category*” (M01); “*racist abuse*” (M06), “*patronising*” (M12); “*really rude and arrogant*” (M05); “*horrible…stigmatising*” (M26); “*a power trip…awful*” (M29). Often what lay behind these descriptions was that staff had belittled or undermined the mothers in some way, at a time when they were particularly vulnerable: “*They were treating me like I was stupid*” (M12) or “*they treated me like I’m acting weird*” (M06).

Some mothers felt powerless to respond to disapproving professionals: “*You don’t want to sort of cause trouble, or be difficult or awkward*” (M14). One described how, when her newborn baby was crying, “*I hadn’t got a clue what I was doing and the midwife was quite like sharp with me. And that didn’t really help*” (M20). This experience had led her to conclude that it was safer not to ask for help: “*You’d better Google rather than midwives… I didn’t want them thinking, ‘Oh, she can’t do it’*” (M20). Another described the power imbalance between herself and professionals and how her confidence in standing up to them had grown between having her first child at age 16 and her second at 20:

“*They talked down to you a lot … You feel quite vulnerable, and then if you’ve got a health professional saying something to you, you’re not going to talk back to them, are you? …[With my second child] I did get a lot of look down… I said, ‘Well, it’s not my problem. I’m married and I’ve got a stable home, if I want to have a child I will.’*” (M36)

Older mothers described similar experiences of being reduced to tears by the insulting or offhand words of staff, and usually did not feel able to complain. One mother with mental health problems described how painful it was to “*just be treated like a weird person ‘cause that’s always what I feel like*” (M29). Another described how poor care undermined her already fragile self-confidence: “*I have had a lot of issues in the past with people telling me I’m not good enough… but that’s exactly what they were doing, making you feel like you weren’t good enough*” (M12).

In the emotionally labile period after birth, even casually thoughtless words could be upsetting for vulnerable mothers. For example, a destitute mother’s distress at learning that she would have to pay thousands of pounds for her maternity care was compounded by the insensitive way this news was delivered: “‘*At the end even [if] you die your children have to pay for your debt.*’ *Yeah, [they] just told me that*” (M11). Another mother gave up when she could no longer cope with persistent unkindness from staff on the postnatal ward: “*I just stopped talking*” (M26).

### 3.3 The importance of choice and control

This theme describes mothers’ wish to remain in control of decisions about their maternity care. Some were very pleased with the way they had been able to exercise informed choice: “*I was in control. Because when I was really in pain the lady came to me and said, ‘If you want epidural I can give you, then you won’t feel the pain,’ and I said, ‘No*’**” (M02). They particularly appreciated it when staff explained the options clearly: “*They give me many choice…everything about that they explained to me*” (M07). Just one mother said that she did not want to make choices, preferring to trust the expertise of the staff: “*I can’t think healthy that time, I can’t make decision… I just leave myself [to] them*” (M10).

Other mothers described situations where their autonomy was not respected, feeling that staff tried to use their power as professionals to pressurise them into making a particular decision. For example, one mother felt bullied into accepting a caesarean, in contrast to a previous experience in her home country where she gave birth naturally after a long labour:

“*[The midwife] didn’t have much patience because after like six, seven hours she was like, ‘They’ll have to do you surgery, they’ll have to do surgery,’ like she’s forcing me to accept that they’ll have to do surgery. I no was happy but, she is the doctor so… At the end she say, ‘No, no, but you have to do it now, you have to do it now.’ I was just say, ‘Okay, give me the form and I sign’*” (M11).

Another mother was distressed that a health visitor used threats to try to enforce her compliance when she mentioned on the new birth visit that she was not planning to vaccinate her baby: “*We don’t want to immunise the child because it is our belief, but they don’t respect it…[the health visitor] was talking about child protection, that she will contact [social services] and then it will be a problem*” (M01).

Some of the mothers were unaware of their right to exercise choices because staff did not tell them. A mother whose baby was in breech presentation was not told about the option of a vaginal breech birth; and another was not initially ‘allowed’ to leave the hospital after birth: “*They told me they won’t let me out until I would do the second thing in the toilet*” (M06). A mother who requested a caesarean because she had two problematic previous labours was refused the right to make this choice: “*I asked my midwife that please can I have a caesarean. And then she said, ‘No, you have to go and see a doctor, the doctor is the one who is going to decide.’...The doctor said, ‘No, you have to have a normal delivery’*”(W032). When her labour did not progress and her baby was delivered by emergency caesarean, she reflected that “*they should have just listened to what I said*”.

Several mothers described their dismay at having painful vaginal examinations during labour, and one (a survivor of human trafficking) described how a midwife “*put their finger in*” without any explanation or attempt to seek consent: “*She did not explain that to me. She just start put - and when I shouted she- she didn’t explain nothing to me. Oh my God!*” (M03). This mother only became aware that she had the right to consent or refuse when a different midwife examined her the next day: “*She explained everything that she wanted to do, she asked to do, it’s not really compulsory for her to do but it’s really good to do it… She explained everything to me and she do it exactly what she asked me*” (M03).

### 3.4 Needing trust to feel safe

This theme describes how some mothers experienced the maternity environment as a place of safety and others as a place of danger, according to the level of trust they had developed in staff. A few of the mothers had inherent confidence in maternity staff because of their status as health professionals: “*They are the professional, I can’t give advice [to] them. They know what to do*” (M08). For others this trust had developed through the quality of care they received: “*I got the best care*” (M39); “*they saved my life*” (M22). Confidence in professionals had led these mothers to feel safe, even where there were obstetric complications: “*A really good service…they take care of both of us*” (M28).

Other mothers were less fortunate. Some described a sense of chaos, particularly on the postnatal ward where no one appeared to be in charge: “*Seems like no-one knew anything… No-one seems to had the idea what’s going on with my case*” (M01). Where professionals gave contradictory information, mothers’ confidence was also undermined: “*The midwives changed every six hours, and everyone says something different*” (M06). For some mothers, poor care was represented not merely by unclear systems and poor information, but by what appeared to be professional ineptitude. For example, they gave accounts of health professionals failing to diagnose placental abruption, or telling a mother that her unusual fetal movements were caused by her eating “*too much chocolate”* (M06). A mother with HIV discovered when she went into labour that the hospital had lost her notes and did not know how to look after her; the doctor was “*dilly-dallying, because she wants the notes*” (M39). This mother was both knowledgeable about prevention of mother-to-child transmission of HIV, and confident enough to be assertive: “*I said, ‘Ask me anything about me, CD4 count, my tablets. You better put up that IV they put for HIV medication’*” (M39).

One mother was very distressed that staff overlooked the red band she was wearing to alert them to serious allergic reactions: “*They nearly gave me antibiotics that I’m seriously allergic to… All written down in my notes. I had a red band on and they still tried to give them to me.*” (M12). She described midwives acting “*like Laurel and Hardy*” as they blamed each other for not having read her notes. The same mother encountered a doctor who was unaware of her health condition, and who reacted scornfully to her attempts to keep herself well:

“*I were drip grey, my veins were closing up, and [the doctor] said, ‘Right, we’ll break your waters now.’ I said, ‘There’s no way you can break my waters now, I need to go on a glucose drip, I’m really quite poorly,’ and he said, ‘Oh, are you a doctor now?’… And I said, ‘No I’m not a doctor, but I have lived with this condition since I were 15,’ and he actually looked at me and said, ‘What condition?’*” (M12)

These experiences left the mother so frightened that she had become hyper-vigilant: “*[Each] time they come in with something I’m like, ‘Who are you? What do you want? What are you doing? What are you giving me?’*” Because she felt she could not trust staff to keep her safe, “*I were convinced I were going to die...I were convinced they were going to kill me*” (M12).

For this mother and several others, poor care was compounded by what they saw as dishonest record keeping: “*They try to blame you* … *it’s actually down in my notes that I refused to take them, not that they tried to give me antibiotics that I’m allergic to*” (M12). Professionals appeared to be unaware how easily they could lose a mother’s trust through misrepresenting the facts, perhaps not realising that the mother could read the notes:

“*I provide urine sample to the midwife, the midwife get my urine sample and open all the cabinet in her office to find something, I think test paper, but she can’t find anything, she poured my urine sample to a sink. Then when I go back home I found she wrote ‘no urine sample’ on my notebook. So I found I cannot trust.*” (M30)

## 4 Discussion

Birth can be an opportunity for mastery experiences that lead to a growth in self-confidence and self-esteem, or alternatively for feelings of failure and humiliation that reduce self-esteem.^28^ The mothers who took part in this study were experiencing multiple disadvantages that could contribute to profound feelings of powerlessness, self-stigmatisation and low self-esteem in their normal lives.^10^, ^29^, ^30^, ^31^ They brought these vulnerabilities to their encounters with maternity professionals, inside what was for most was a confusing and sometimes frightening system which they did not fully understand, due to barriers of confidence as well as language.^7^, ^11^, ^13^, ^32^

The attitude of maternity professionals was crucial in determining whether mothers’ vulnerability was increased or moderated.^33^, ^34^ Where mothers were received with warmth, kindness and respect, their self-esteem grew and they flourished.^11^, ^33^ Professionals who really listened to mothers gave them the sense that they were seen “*as a person*” and worthy of this attention, which helped to overcome internalised feelings of low self-worth or being “*less than anybody else*”*.*^34^, ^35^ McGill-Cuerden challenges midwives to understand maternity service users as “guests of the hospital staff” who should be treated accordingly,^36^ and there were examples in this study of mothers being made to feel welcome, safe, and even “*special*”*.*

By contrast, some mothers were processed through an impersonal system with little explanation or attention to their needs, and staff appeared not to realise the extent of their need for information about the purpose and mechanics of the maternity system and the progress of their pregnancies. Mothers were made to feel “*low category*”, “*stupid*” or “*weird*” by professionals who were rude, judgemental, thoughtless or hasty. This further undermined their already low self-confidence^33^ and left them demoralised and tending to withdraw from further interactions. Some experienced the maternity environment as a place of chaos and potential danger because health professionals had not demonstrated competence, consistency or trustworthiness.^37^ It is also important to acknowledge that some of the mothers showed great resilience in the face of the stresses in their lives.^38^ These mothers brought a degree of assertiveness to difficult encounters with health professionals and stood up for themselves, trying to establish their right to be taken seriously as experts about themselves.^37^

For women who often had little control over other aspects of their lives, it was of great significance to have some control over what was done to their bodies. Some were offered the right to make choices over care and supported appropriately to make meaningful informed choices; others were denied choices or felt bullied into accepting what the professional wanted.^39^ It is inevitably difficult for more vulnerable mothers to have the confidence to disagree with professionals’ recommendations or assertions.^37^ The right to accept or decline an invasive procedure such as a vaginal examination is important to all women, but failure to explain the procedure or to obtain consent may be particularly traumatic for survivors of sexual violence.^40^

The mothers in this study were not specifically asked about how care they received was organised. However, many spontaneously described receiving care either from a large number of midwives, or alternatively from one specialist midwife or small team. (The term ‘specialist’ is used here in the sense that the mothers themselves used it, and it is not known whether these midwives were also strategic leaders who would meet the Royal College of Midwives’ definition of a ‘specialist midwife’.^41^) Some mothers were satisfied with receiving care from many maternity professionals if the professionals all had positive and helpful attitudes. However, more commonly mothers reported encounters with professionals who had a range of attitudes and interpersonal skills, including negative ones. Furthermore, where there were many different midwives involved, the ‘outsider’ status of vulnerable women was not recognised and no one took responsibility for explaining how the system worked. Volunteer peer supporters and doulas can have a valuable role in explaining the maternity system and supporting mothers to access it, but these third sector projects currently only reach small numbers of women.^26^, ^42^

The women who had the most consistently positive experiences were those receiving specialist care. On a practical level, the specialist midwives recognised their confusion and fears and helped to guide women through the unfamiliar maternity system and procedures. On an emotional level, the continuity of care enabled relationships of trust to develop with even the most vulnerable mothers, and the increased time that midwives had spent with the mothers enabled them to address the women’s complex needs more fully. Although women generally prioritise kindness and professional competence over continuity,^43^ continuity of care midwifery models can improve outcomes including for mothers with complex social factors.^44^ This study illustrates how personalised, relationship-based maternity care can transform highly vulnerable mothers’ experiences of care, a finding which is consistent with other studies of disadvantaged mothers.^35^, ^45^

‘Dignity’ and ‘confidence’ are key aspects of women’s concepts of ‘integrity’ in healthcare.^34^ Mothers experiencing multiple disadvantages have the same desire as other mothers to be treated with kindness, courtesy and “as a human being” by maternity providers,^46^ and respectful, non-abusive, consent-based treatment during birth can also be considered a human right.^47^ Guidelines from the National Institute of Health and Care Excellence (NICE) urge maternity professionals to treat all mothers with kindness, respect, dignity and compassion, ensuring that the mother remains in control and that she is able to make informed choices; their guidelines also specify that mothers with ‘complex social factors’ should be given the information and support they need to understand their pregnancy and access services.^1^, ^22^ This research exposes the gap that exists between these aspirations and the actual experiences of many vulnerable mothers in England.

A key strength of this research that by working with a range of third sector organisations supporting disadvantaged mothers in various parts of England, we were able to reach very vulnerable women, with diverse experiences of multiple disadvantages, whose voices are not typically heard in maternity services research. It was a limitation that none of the women took up the offer of professional interpreting, as this limited the depth of a few of the interviews.

## 5 Conclusion

Mothers experiencing multiple disadvantages bring pre-existing vulnerabilities into their encounters with maternity services, which affect their experiences of maternity care. Like other mothers, they value being treated as an individual, making informed choices, and feeling safe. They may, however, lack the confidence to assert their rights to dignified treatment, and their sense of powerlessness and low self-esteem can be significantly worsened by disrespectful care. Training and supervision should enable maternity professionals to understand how confusing maternity care can be to very disadvantaged mothers, emphasising the need to provide clear, accessible and empowering information and guidance to enable all mothers to make choices and navigate through the system. Non-clinical staff and peer supporters could have a role in supporting and guiding vulnerable mothers through their maternity care. This research supports the value of specialist midwives, using a relationship-based approach, in delivering a high quality service to mothers experiencing multiple disadvantages, but also shows that leaders of maternity services need to do more to challenge negative staff attitudes across the board and ensure that that all mothers, irrespective of social complexity, are treated at all times with kindness, respect and dignity.

## Funding

This paper reports on an independent study which is funded by the Policy Research Programme in the Department of Health. The views expressed are not necessarily those of the Department.

## Competing interests

The authors declare they have no competing interests.

## Author contributions

This study is part of a programme of work, the research questions for which were developed by MR and JM. MR and JM conceived and developed the outline for this study. JM undertook the data collection and JM and MR both took part in data analysis. JM drafted the manuscript with input from MR. Both authors were involved in interpretation, review and revision of the draft manuscript and approval of the final version.

## Ethics approval and consent to participate

The University of Oxford Medical Sciences Ethics Committee (reference MSD-IDREC-C1-2013-111) approved the study on 14th July 2013. An information leaflet was provided and written informed consent to participate was obtained. Women consented to data collection and for their experiences to be used in reports or publications with no details or other information being published that could identify them. Following the consent process the individual qualitative interview transcripts will not be made publicly available.
